# Avoiding Postnatal Growth Retardation by Individualized Fortification of Breast Milk: Implications for Somatic and Neurodevelopmental Outcomes

**DOI:** 10.1089/bfm.2019.0031

**Published:** 2019-04-17

**Authors:** Christoph Fusch

**Affiliations:** Department of Pediatrics, Nuremberg General Hospital, Paracelsus Medical University, Nuremberg, Germany.

**Keywords:** human milk, fortification, variation

The American Academy of Pediatrics recommends that preterm infant growth should imitate fetal growth, yet the postbirth growth trajectories for most preterm infants differ from the normal intrauterine trajectory (Fig. 1).^1,2^ Preterm infants have high growth rates and thus high nutritional needs. Optimal growth requires a delicate balance between protein, energy, carbohydrates, and fat. Inadequate nutrition early in development can restrict growth and alter body composition, potentially leading to increased risk of disease during adulthood (i.e., the “developmental origins of health and disease” hypothesis).^2^ Postnatal growth retardation is a uniform problem among preterm infants, suggesting that current feeding practices are not sufficient to meet the nutritional needs of these infants.

**FIG. 1.**
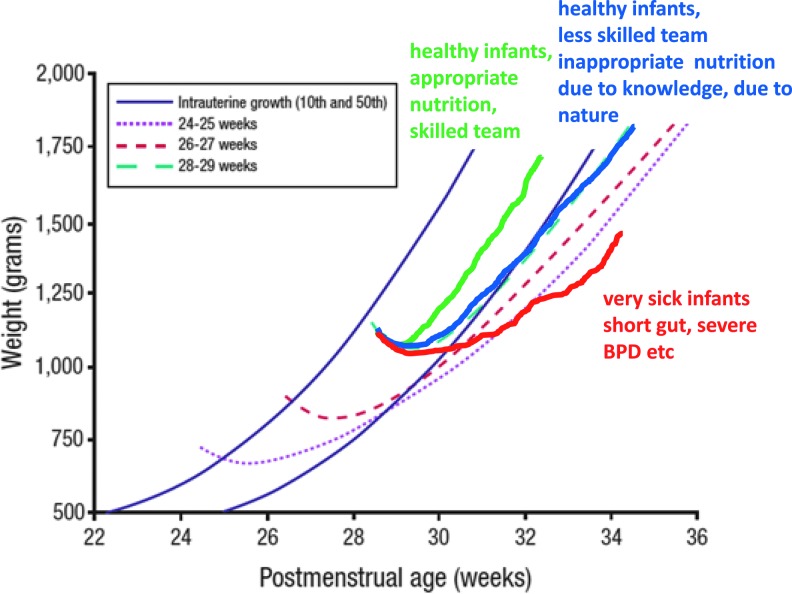
Postnatal growth restriction in very low birth weight infants. Adapted from Ehrenkranz et al., 1999.^1^

Breast milk is routinely fortified with fixed dosages of human- or bovine-based fortifiers to meet the nutritional demands of preterm infants. However, there is considerable inter- and intraindividual variation in protein, carbohydrate, and fat content of breast milk that may potentially impact energy and nutrient intake, as the exact macronutrient content is unknown (Fig. 2).^3,4^ These variations in macronutrient content may have significant impacts on infant growth and body composition. It has been estimated that ∼60% of very low birth weight infants fed fortified breast milk have postnatal growth restriction.^5^ Inadequate nutrition in preterm infants may also impact longer term neurodevelopmental outcomes. A study in extremely low birth weight infants found that each kcal/kg per day of energy intake during the first week of life was associated with a 0.46-point increase on the Bayley Mental Development Index (MDI) at 18 months; each gram/kg per day of protein intake increased Bayley MDI scores by 8.2 points.^6^

**FIG. 2.**
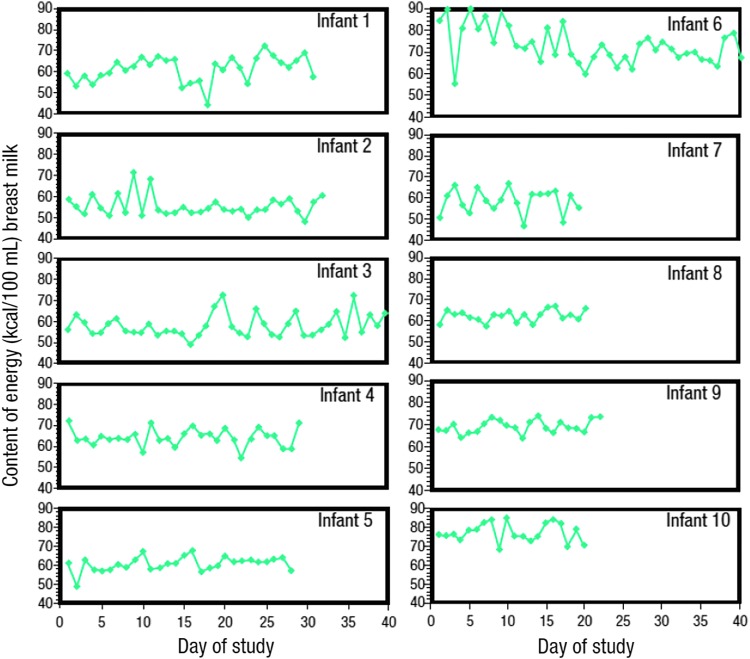
Inter- and intraindividual variation in breast milk samples.

Individualized fortification of breast milk is a new strategy that may help to optimize nutrition for preterm infants. This approach involves analyzing breast milk and individually fortifying it to reach recommended macronutrient amounts, with the goal of standardizing intake for preterm infants. Many studies have shown that the macronutrient content (i.e., protein, fat, and carbohydrates) of human milk can be rapidly measured using near-infrared milk analyzers; however, additional studies are needed before they can be used in clinical practice.^7^ Accurate measurements will also be dependent on adherence to good laboratory and clinical practice in the calibration and validation of the instruments, as well as appropriate sample preparation.^8^ An international multicenter study (MAMAS) is currently assessing the performance of bedside milk analysis.

A small pilot study in healthy very low birth weight infants demonstrated the safety and feasibility of individualized fortification of breast milk.^3^ A linear relationship between milk intake and weight gain was seen in infants fed fortified milk for 3 weeks (*n* = 10), whereas growth rates in infants fed standard fortified milk (*n* = 20) seemed to be independent of milk intake (Fig. 3a). Preliminary findings from a larger randomized controlled trial evaluating individualized target fortification in preterm infants suggest that individualized fortification improved intake of fat, protein, and carbohydrates compared with standard fortification.^9^ Target fortification also increased caloric intake and improved growth outcomes, such as weight gain and growth velocity. The impact on growth was most pronounced in a subgroup of infants who received breast milk with a lower protein content than average (Fig. 3b). Although not significant, there was a trend toward improvement in neurodevelopmental outcomes in the targeted fortification group, with an effect size of the same magnitude observed in other studies on nutritional intake and IQ.

**FIG. 3.**
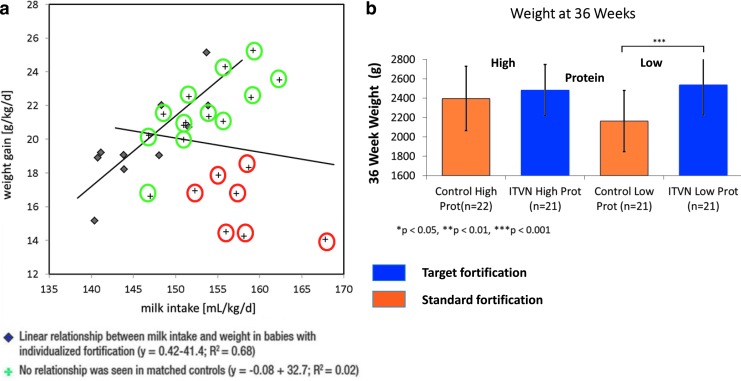
Target fortification of breast milk leads to predictable growth **(a,b)**. **3a** is modified from Rochow et al., 2013.

Although breast milk is best from a biological (availability/digestibility/holistic) perspective, it may provide a highly unstandardized diet for preterm infants who are not self-regulated and can put growth-associated outcomes at risk. Individualized target fortification of breast milk is feasible and seems to have a positive impact on growth and other outcomes in preterm infants. Further randomized controlled trials are needed to determine the optimum components for target fortification and to assess effects on body composition and long-term outcomes.

## References

[1] Ehrenkranz RA, Younes N, Lemons JA, et al. Longitudinal growth of hospitalized very low birth weight infants. Pediatrics 1999;104:280–289 10429008 10.1542/peds.104.2.280

[2] Rochow N, Raja P, Liu K, et al. Physiological adjustment to postnatal growth trajectories in healthy preterm infants. Pediatr Res 2016;79:870–879 26859363 10.1038/pr.2016.15

[3] Rochow N, Fusch G, Choi A, et al. Target fortification of breast milk with fat, protein, and carbohydrates for preterm infants. J Pediatr 2013;163:1001–1007 23769498 10.1016/j.jpeds.2013.04.052

[4] Sauer CW, Boutin MA, Kim JH Wide variability in caloric density of expressed human milk can lead to major underestimation or overestimation of nutrient content. J Hum Lact 2017;33:341–350 28418793 10.1177/0890334416672200

[5] Henriksen C, Westerberg AC, Rønnestad A, et al. Growth and nutrient intake among very-low-birth-weight infants fed fortified human milk during hospitalisation. Br J Nutr 2009;102:1179–1186 19445820 10.1017/S0007114509371755

[6] Stephens BE, Walden RV, Gargus RA, et al. First-week protein and energy intakes are associated with 18-month developmental outcomes in extremely low birth weight infants. Pediatrics 2009;123:1337–1343 19403500 10.1542/peds.2008-0211

[7] Fusch G, Kwan C, Kotrri G, et al. “Bed side” human milk analysis in the neonatal intensive care unit: A systematic review. Clin Perinatol 2017;44:209–267 28159207 10.1016/j.clp.2016.11.001

[8] Fusch G, Kwan C, Huang RC, et al. Need of quality control programme when using near-infrared human milk analyzers. Acta Paediatr 2016;105:324–325 26663457 10.1111/apa.13305

[9] Rochow N, Fusch G, Ali A, et al. Target Fortification with Protein, Lactose and Fat for Preterm Infants Improves Growth Outcomes - A Double-Blind Randomized Controlled Trial. Annual Meeting of the Pediatric Academic Society (PAS), 2016, Baltimore

